# Supplementary material for: Experiences, challenges, and enablers for promoting interprofessional education among medical students: A scoping review
Source: PLoS One. 2026 Jun 1;21(6):e0331157. doi: 10.1371/journal.pone.0331157 (PMC13225340; doi:10.1371/journal.pone.0331157)
Supplement: S2 File — (DOCX) [file pone.0331157.s002.docx]

**Information Sources**

A comprehensive literature search was conducted across three electronic databases: PubMed, Scopus, and Web of Science. These databases were selected because they index a broad range of peer-reviewed literature in health professions education, medical education, and interprofessional education.

The search covered studies published between January 2014 and December 2024 and was limited to articles written in English.

The search aimed to identify studies exploring interprofessional education (IPE) among medical students and health professions students, including their experiences, perceptions, challenges, and enabling factors.

All retrieved records were exported into EndNote reference management software, where duplicates were removed prior to screening.

**Search Strategies**

PubMed

("interprofessional education" OR "interprofessional learning" OR "interprofessional collaboration" OR "interprofessional practice") AND ("medical students" OR "health professions students" OR "healthcare students") AND ("experience" OR "perception" OR "attitude" OR "barrier" OR "challenge" OR "facilitator")

Filters applied: Publication years 2014–2024; Language: English.

Scopus

TITLE-ABS-KEY ("interprofessional education" OR "interprofessional learning" OR "interprofessional collaboration") AND TITLE-ABS-KEY ("medical students" OR "health professions students") AND TITLE-ABS-KEY ("experience" OR "perception" OR "barrier" OR "challenge" OR "facilitator")

Filters applied: Document type Articles; Language English; Year 2014–2024.

Web of Science

TS= ("interprofessional education" OR "interprofessional learning" OR "interprofessional collaboration") AND TS= ("medical students" OR "health professions students") AND TS=("experience" OR "perception" OR "barrier" OR "challenge" OR "facilitator")

Filters applied: Publication years 2014–2024; Language: English.

Study Identification

The database searches produced approximately 1,500 records, which were exported to EndNote for duplicate removal and screening. The study selection process followed PRISMA-ScR guidelines and is illustrated in the PRISMA flow diagram (Figure 1) in the manuscript.
