# Supplementary material for: Experiences, challenges, and enablers for promoting interprofessional education among medical students: A scoping review
Source: PLoS One. 2026 Jun 1;21(6):e0331157. doi: 10.1371/journal.pone.0331157 (PMC13225340; doi:10.1371/journal.pone.0331157)
Supplement: S3 File — (DOCX) [file pone.0331157.s003.docx]

**Table 1. Characteristics of the included studies (n = 8)**

| S/N | Authors (Year) | Country | Objective | Study Design | Sample Size | Participants | IPE Intervention | Measures | Analysis | Key Findings |
| --- | --- | --- | --- | --- | --- | --- | --- | --- | --- | --- |
| 1 | Ernawati & Utami (2020) | Indonesia | To evaluate the impact of IPE programme on collaborative competencies. | Pre–post study | 138 | Health students (incl. medical) | Community-based IPE involving lectures, group discussions, and fieldwork (one semester analysed) | ICCAS | Paired-sample t-test (collaboration: p=0.001; communication: p>0.05) | Reduced collaboration scores; no significant change in communication |
| 2 | Thompson et al. (2020) | United Kingdom | To assess readiness for IPE before and after geriatric IPE sessions. | Controlled before–after study | 300 medical and 150 nursing students (with partial response rates) | Undergraduate medical and nursing students at different training levels | Case-based geriatric PBL sessions with facilitated group discussion and collaborative learning | Modified RIPLS questionnaire and open-ended qualitative responses | Wilcoxon signed-rank test (with additional subgroup t-tests) | Improved teamwork and professional identity; reduced hierarchy concerns; no change in role understanding |
| 3 | Xing et al. (2024) | China | To explore medical students’ lived experiences of IPE. | Descriptive qualitative study (Phenomenological approach) | 10 | Undergraduate medical students | Structured 2-week nursing internship (early clinical IPE exposure) | Semi-structured interviews (Colaizzi method) | Not applicable | Positive experiences of collaboration and engagement; improved role awareness; time-related constraints reported |
| 4 | Berger-Estilita et al. (2020) | Switzerland | To examine attitudes toward IPE and determine optimal timing within the curriculum. | Sequential explanatory mixed-methods design | Survey=683 Interviews=31 | Medical students (Years 1–6) | Optional and compulsory IPE activities embedded in the curriculum (e.g., internships, seminars, skills training) | G-IPAS | Inferential statistics (ANOVA, t-tests; p-values reported) | Positive attitudes toward IPE; support for early integration; barriers from stereotypes and curriculum structure |
| 5 | Alzamil & Meo (2020) | Saudi Arabia | To evaluate medical students’ readiness and perceptions regarding IPE implementation. | Cross-sectional survey | 158 | Undergraduate medical students (including interns where specified) | No specific intervention (survey of IPE perceptions) | RIPLS (19-item scale) | Descriptive statistics (percentages, means; p-values reported) | Positive perception of IPE; readiness for shared learning; role uncertainty noted |
| 6 | Al-Qallaf et al. (2024) | Bahrain | To explore attitudes, expectations, and contextual factors influencing IPE adoption. | Qualitative study using thematic analysis (grounded theory approach) | 16 | Medical and nursing students (class representatives across levels) | No intervention (exploration of IPE perceptions through FGDs and interviews) | Focus group discussions and interviews (thematic analysis) | Not applicable | Experiences shaped by role recognition, team dynamics, and cultural context; influence of social and organisational factors |
| 7 | Zanotti et al. (2015) | Italy | To assess the effectiveness of on-field IPE training on medical students’ attitudes. | Pre–post study | 277 (completed pre–post; from 421 enrolled) | Second-year medical students | Observation-based and practice-based clinical IPE training | IEPS | Paired t-test; Wilcoxon signed-rank test | Improved attitudes toward teamwork; stronger gains in competence and cooperation; limited change in value understanding |
| 8 | Prentice et al. (2015) | Canada | To understand medical and nursing students ‘experiences of collaboration. | Phenomenological  Study | 17 | Medical (Years 1–2) and nursing students (Years 3–4) | No specific intervention(exploration of experiences from prior IPE activities including workshops and simulations) | Conversational interviews (face-to-face, semi-structured) | Not applicable | Experiences influenced by professional identity and prior perceptions; need for interaction highlighted |
