# Supplementary material for: Experiences, challenges, and enablers for promoting interprofessional education among medical students: A scoping review
Source: PLoS One. 2026 Jun 1;21(6):e0331157. doi: 10.1371/journal.pone.0331157 (PMC13225340; doi:10.1371/journal.pone.0331157)
Supplement: Attachment — Submitted filename: RESPONSE TO REVIEWERS-SCOPING REVIEW.docx [file pone.0331157.s005.docx]

# Response to Reviewers

Manuscript ID: PONE-D-25-43439

Title: Experiences, challenges, and enablers of IPE among medical students: A scoping review

Journal: PLOS ONE

Dear Editor and Reviewers,

We sincerely thank the Academic Editor and the reviewers for their constructive and insightful comments on our manuscript.

We greatly appreciate the time and effort invested in evaluating our work. We have carefully revised the manuscript in response

to all comments and suggestions.

All revisions have been incorporated into the manuscript. A tracked‑changes version and a clean revised manuscript have been

Submitted as requested.

Below, we provide a detailed, point‑by‑point response to each comment.

**EDITOR COMMENTS**

Comment: Define “interprofessional education (IPE)” at its first appearance.

Response:

Thank you for this suggestion. We have revised the introduction to clearly define interprofessional education (IPE) at its

first occurrence and ensured that the abbreviation IPE is used consistently throughout the manuscript.

Location: Introduction section, paragraph 1.

Comment: Review and correct all in‑text citations and reference formatting to match PLOS ONE style.

Response:

All references and in‑text citations have been reviewed and corrected to comply with PLOS ONE referencing guidelines, including

author formatting, journal titles, issue numbers, page ranges, and DOIs.

Comment: Improve clarity and remove fragmented sentences.

Response:

The manuscript has undergone thorough language editing to improve clarity, remove fragmented sentences, and ensure consistency in academic writing.

**METHODOLOGICAL COMMENTS**

Comment: Expand the Methods section and clarify search strategy and inclusion criteria.

Response:

The Methods section has been expanded substantially. Additional details are now provided regarding:

1. Databases searched
2. Search terms and Boolean operators
3. Inclusion and exclusion criteria
4. Screening and study selection process
5. Data extraction and thematic synthesis procedures
6. Full search strategies for each database are included as Supplementary File 2.

Comment: Clarify the search period (2014–2024).

Response:

A justification has been added explaining that the search period reflects contemporary IPE practice and curriculum development

in health professions education over the past decade. We also clarified that no studies published between 2014 and 2018 met

the inclusion criteria after full‑text screening.

**PRISMA FLOW AND REPORTING**

Comment: Correct inconsistencies between the PRISMA diagram and text.

Response:

We carefully reviewed the screening process and corrected inconsistencies between the PRISMA diagram and the manuscript text.

All numerical values now match exactly.

Corrected values:

Records identified: 1500

Duplicate records removed: 1288

Records screened: 212

Full‑text articles assessed: 50

Studies included: 8

The corrected PRISMA 2020 flow diagram is included as Figure 1.

Comment: Follow PRISMA‑ScR guidelines.

Response:

The manuscript now fully adheres to PRISMA‑ScR reporting guidelines. The following supplementary files have been included:

1. Supplementary File 1 – PRISMA‑ScR checklist
2. Supplementary File 2 – Full search strategies
3. Supplementary File 3 – Data charting template

**RESULTS AND DISCUSSION**

Comment: Present narrative results before tables and figures.

Response:

The Results section has been reorganized so that narrative findings are presented first, followed by tables summarizing the

Included studies.

Comment: Strengthen the discussion and connect findings to literature.

Response:

The Discussion section has been expanded to include additional recent literature on interprofessional education, especially

regarding collaborative practice, professional identity formation, and experiential learning in medical education.

**DATA AVAILABILITY**

Comment: Ensure compliance with PLOS ONE data availability policy.

Response:

The Data Availability Statement has been revised. All data underlying the findings are available within the manuscript and

Supporting information files, including search strategies, PRISMA checklist, and data charting template.

**TECHNICAL AND FORMATTING CORRECTIONS**

Additional revisions made:

1. Corrected grammatical and typographical errors throughout the manuscript
2. Ensured tables and figures are numbered sequentially and cited in the text
3. Removed color highlights from the PRISMA diagram
4. Ensured references follow PLOS ONE formatting requirements

RESPONSES TO REVIEWER 1

Comment: Define IPE before using the abbreviation.

Response:

The term interprofessional education (IPE) is now defined at its first occurrence in the Introduction section.

Comment: PRISMA diagram inconsistency.

Response:

The PRISMA diagram and numerical values reported in the Results section have been corrected to ensure full consistency.

RESPONSES TO REVIEWER 2

Comment: Manuscript contains fragmented or unclear sentences.

Response:

The manuscript has been thoroughly edited to correct grammatical errors and improve clarity.

Comment: Assertions without citations.

Response:

Additional references have been added throughout the Introduction and Discussion sections to support statements about the

existing literature.

Comment: Methods section unclear.

Response:

The Methods section has been substantially expanded to improve transparency and reproducibility, including detailed descriptions

of search strategy, study selection, data charting, and thematic synthesis.

We thank the editor and reviewers again for their valuable feedback, which helped improve the quality and clarity of the

Manuscript. We hope the revised manuscript now satisfactorily addresses all concerns raised during peer review.

Sincerely,

Maxwell Ateni Assibi

Corresponding Author

Department of Health Professions Education and Innovative Learning

University for Development Studies

Email: assibimaxwell93@gmail.com
