# Supplementary material for: Experiences, challenges, and enablers for promoting interprofessional education among medical students: A scoping review
Source: PLoS One. 2026 Jun 1;21(6):e0331157. doi: 10.1371/journal.pone.0331157 (PMC13225340; doi:10.1371/journal.pone.0331157)
Supplement: Attachment — Submitted filename: Response To Reviewers Letter(3).docx [file pone.0331157.s006.docx]

**Manuscript ID:** PONE-D-25-43439R1
**Title:** Experiences, challenges, and enablers for promoting interprofessional education among

Medical students: A scoping review

Response to Academic Editor and Reviewers

Dear Editorial Team,

We sincerely thank you for the careful evaluation of our manuscript and for the constructive and insightful feedback provided. We are grateful for the positive assessment of our work and the recognition of its contribution. We have carefully revised the manuscript to address all comments raised and believe that these revisions have significantly improved the clarity, rigor, and overall quality of the paper.

Below, we provide a detailed, point-by-point response to all editorial comments.

Editorial Comments and Responses

**Comment 1: Strengthen the Discussion (Priority)**

*The discussion is informative but remains partially descriptive. Emphasize interpretation, explain what findings mean in practice, and strengthen linkage to implications.*

**Response:**
We thank the Editor for this important suggestion. The Discussion section has been substantially revised to improve interpretive depth and reduce descriptive repetition.

1. We have strengthened the interpretation of findings by clearly explaining their implications for medical education practice.

2. Explicit statements have been added to address what the findings change and why they matter.

3. Stronger connections between findings and practical implications have been incorporated, particularly in Sections 4.1 and 4.4.

These revisions ensure that the Discussion now focuses more on critical interpretation rather than description.

**Comment 2: Sharpen the Conclusion**

*The conclusion should be more focused and impactful.*

**Response:**
The Conclusion section has been revised to improve clarity, conciseness, and impact.

1. Redundant statements have been removed.

2. The conclusion now clearly highlights:

- The main contribution of the study
- The key implication for practice
- A forward-looking research direction

The revised conclusion is now more synthesis-driven and aligned with the Editor’s expectations.

**Comment 3: Improve Flow and Reduce Repetition**

*There is redundancy across Results and Discussion.*

**Response:**
We have carefully revised the manuscript to improve flow and eliminate redundancy.

1. Repetitive content has been removed from the Discussion section.

2. The Results section now focuses strictly on presenting findings.

3. The Discussion has been refined to focus on interpretation and implications.

This has improved the overall coherence and readability of the manuscript.

**Comment 4: Consistency and Clarity**

*Ensure consistent terminology, alignment of dates/inclusion criteria, and improve language clarity.*

**Response:**
We have thoroughly reviewed the manuscript to ensure consistency and clarity.

1. Terminology has been standardized throughout the manuscript.

2. Dates and inclusion criteria (2014–2024) have been checked and aligned.

3. Minor language edits have been made to improve clarity and readability.

4. Typographical and grammatical inconsistencies have been corrected.

**Comment 5: Reference List Accuracy**

**Response:**
The reference list has been carefully reviewed and updated to ensure accuracy, completeness, and consistency with journal guidelines. No retracted articles were identified. Minor formatting corrections were made where necessary.

We appreciate the Editor’s and Reviewers’ valuable feedback, which has helped improve our manuscript. We hope that the revised version meets the journal’s requirements and is suitable for publication in PLOS ONE.

**Sincerely,**
Maxwell Ateni Assibi
(On behalf of all authors)
